# Current Synthetic Routes to Peptidyl Mono-Fluoromethyl Ketones (FMKs) and Their Applications

**DOI:** 10.3390/molecules25235601

**Published:** 2020-11-28

**Authors:** Carissa M. Lloyd, Neil Colgin, Steven L. Cobb

**Affiliations:** 1 Department of Chemistry, Faculty of Science, Durham University, Durham DH1 3LE, UK; carissa.m.lloyd@durham.ac.uk; 2 Cambridge Research Biochemicals, 17-18 Belasis Court, Belasis Hall Technology Park, Billingham, Cleveland TS23 4AZ, UK; Neil.Colgin@crbdiscovery.com

**Keywords:** peptide, fluoromethyl ketone, peptide synthesis, peptide modification, fluorine, therapeutic, probe

## Abstract

Peptidyl mono-fluoromethyl ketones (FMKs) are a class of biologically active molecules that show potential as both protease inhibitors for the treatment of a range of diseases and as chemical probes for the interrogation of cellular processes. This review describes the current solid- and solution-phase routes employed for the synthesis of peptidyl mono-FMKs. In addition, it provides a brief overview of some of the key applications of FMKs in the fields of chemical biology and medicinal chemistry.

## 1. Introduction

**Figure 1:**
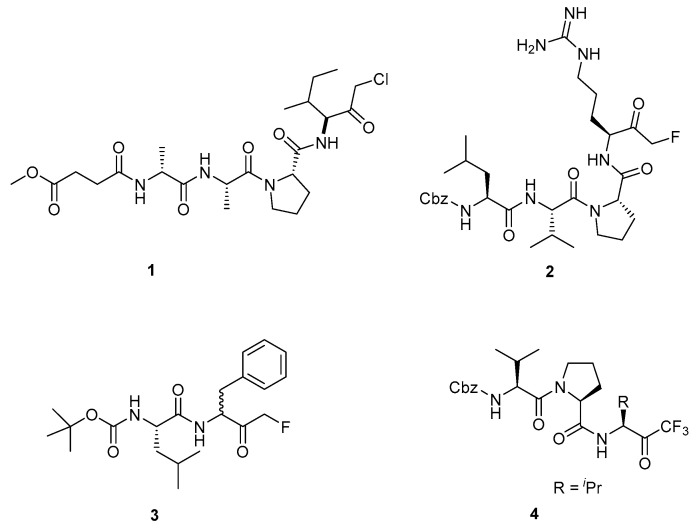
Representative examples of a peptidyl chloromethyl ketone (**1**) [12], peptidyl fluoromethyl ketones (**2** [3] and **3** [2]), and a peptidyl tri-fluoromethyl ketone (**4**) [15].

**Figure 2:**
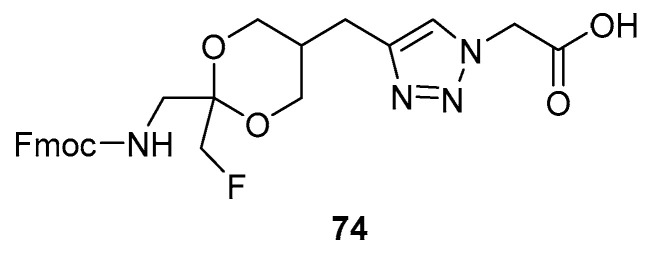
FMK moiety bound to linker for solid-phase peptide synthesis (SPPS) [31].

**Figure 3:**
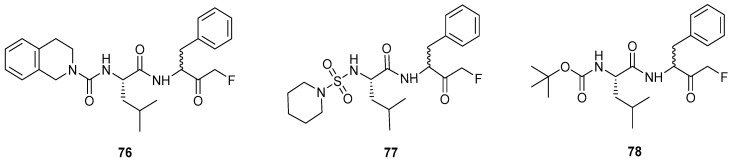
Peptidyl FMKs capable of inhibiting calpain I [2].

**Figure 4:**
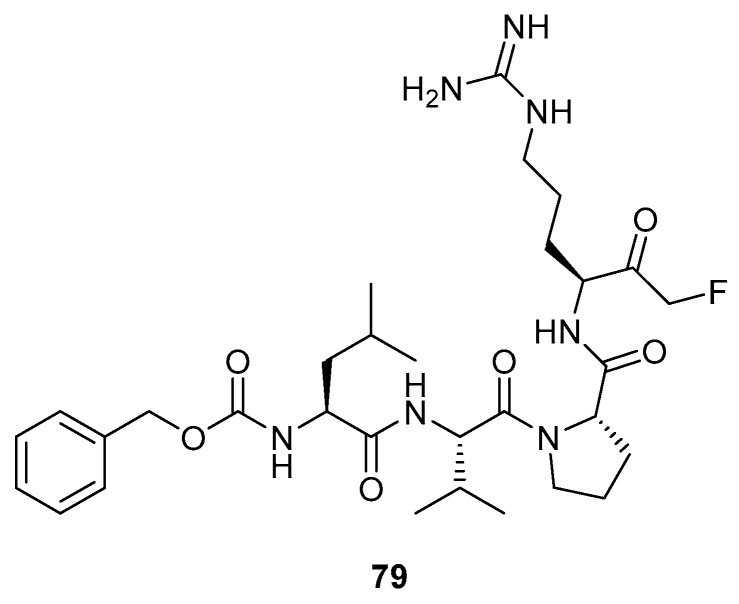
Peptidyl FMK capable of inhibiting the paracaspase enzyme MALT1 [3].

Peptidyl mono-fluoromethyl ketones (FMKs) are a class of biologically active compounds that have been developed as protease inhibitors [1,2,3,4,5] and as chemical probes for the interrogation of cellular processes [6,7,8]. Peptidyl FMKs were first reported in 1985 [9] and have since gained preference in the field over the corresponding peptidyl chloromethyl ketones (CMKs), largely due to their lower reactivity towards nucleophiles. For example, peptidyl CMKs have been developed as both cysteine and serine protease inhibitors (e.g., Figure 1, (**1**)), but their highly reactive nature often causes issues with regards to selectivity. As such, for many peptidyl CMKs, indiscriminate binding of nonproteolytic enzymes leads to undesirable side-effects, rendering them unsuitable for in vivo applications [10]. Peptidyl FMKs are much less prone to nonspecific alkylations due to the intrinsic strength of the C-F bond making them significantly more selective [9] (Figure 1, (**2**), (**3**)). Since their discovery, the number of synthetic routes that are available to access peptidyl mono-FMKs remains rather limited, particularly when compared to those that can be used to access the corresponding peptidyl CMKs [11,12,13] and even peptidyl tri-fluoromethyl ketone systems (Figure 1, (**4**)) [14,15,16].

This review provides an overview of the current state-of-the-art with regards to solid- and solution-phase synthetic routes that can be employed for the synthesis of peptidyl mono-FMKs. In addition, a brief summary of the key applications of this class of compound within the fields of chemical biology and medicinal chemistry is given. For a more detailed overview of FMKs within the field of medicinal chemistry, an excellent recent review of this area was published by A. Citarella and N. Micale [17].

## 2. Current Synthetic Routes to Peptidyl Mono-FMKs

### 2.1. Solution-Phase Routes

#### 2.1.1. Halogen-Exchange

**Scheme 1:**
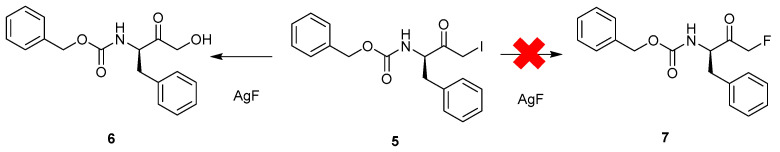
Attempted formation of a peptidyl mono-fluoromethyl ketone (FMK) (**7**) through a halogen-exchange reaction [9].

An early attempted solution-phase peptidyl mono-FMK synthesis looked to use the displacement of bromide or chloride with a source of inorganic fluoride in a halogen-exchange reaction. However, this approach did not produce the desired FMK (**7**), but rather, resulted in a nonfluorinated compound (**6**) along with various unwanted side-products (Scheme 1) [9].

**Scheme 2:**
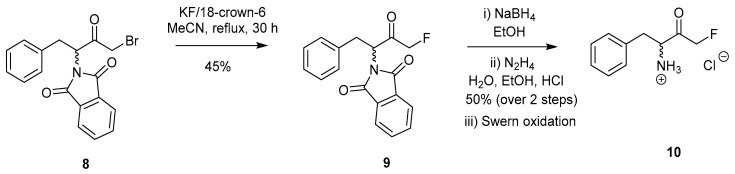
Formation of FMK **10** via a halogen-exchange reaction [18].

Since then, a successful halogen-exchange method has been developed and was reported by Kolb et al. in 1986 (Scheme 2) [18]. Initial isolation of 3-phthalimido-1-bromo-4-phenyl-2-butanone (**8**) from *N*-phthaloyl phenylalanine was achieved through generation of the corresponding diazoketone via the acid chloride (not shown). Subsequent bromination of the diazoketone according to previously known conditions [19,20] led to the formation of **8**. Further reaction of **8** with KF/l8-crown-6 led to FMK **9** in a 45% yield (Scheme 2). Reduction by sodium borohydride enabled removal of the phthaloyl protecting group, however, this simultaneously reduced the ketone functionality which had to be reinstalled by Swern oxidation (**10**) [21,22]. Vederas et al. utilised a similar approach for the synthesis of a ^13^C-labelled peptidyl FMK probe which was used to investigate the mode of action of the HAV 3C enzyme [23].

#### 2.1.2. Synthesis via Direct Fluorination of a Diazo Intermediate

**Scheme 3:**
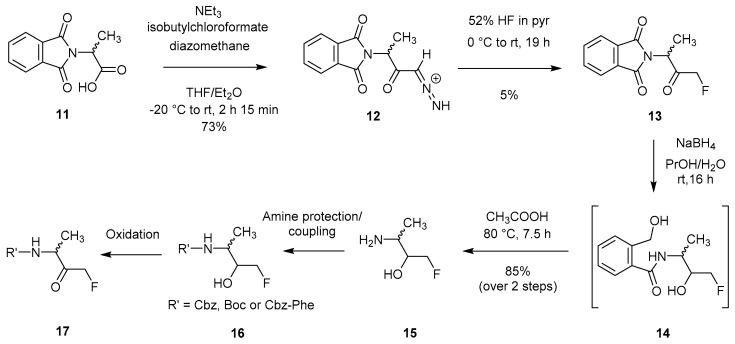
Formation of peptidyl mono-FMK building block (**17**) via a diazo intermediate [24].

Another initially unsuccessful attempt involved the reaction of HF with a diazomethyl ketone; a method analogous to that used for the synthesis of CMKs (chloromethyl ketones) and BMKs (bromomethyl ketones). This reaction, which was attempted on Cbz-Phe-CHN_2_, did not yield the desired FMK, but instead resulted in the formation of the cyclic product 1-oxa-3-aza-4-benzylcyclohexan-2,5-dione [9,24]. It was found that the use of HF/pyridine [25] allowed the successful isolation of the desired product. Furthermore, the use of phthaloyl instead of Cbz as a protecting group also ensured that no amide proton was present, thus preventing unwanted internal cyclisation. Generation of the diazo compound (**12**) was first achieved through the reaction of **11** with diazomethane in the presence of triethylamine and isobutylchloroformate (Scheme 3). Subsequent exposure to HF/pyridine led to the isolation of FMK **13**. Removal of the phthaloyl group under reducing conditions allowed peptide chain extension [24], however, the FMK carbonyl functionality needed to be regenerated in the final step through oxidation.

#### 2.1.3. Dakin–West Modification

**Scheme 4:**
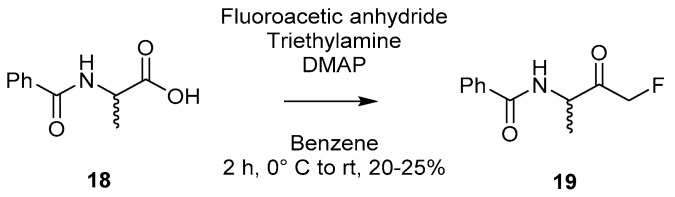
Formation of peptidyl mono-FMK building block Bz-DL-Ala-CH_2_F (**19**) via a modified Dakin–West reaction [9].

A commonly used approach for the synthesis of keto-amides, the Dakin–West reaction [26], was modified [9] in order to allow for the synthesis of a selection of peptidyl-FMKs, as shown in Scheme 4 for Bz-DL-Ala-CH_2_F (**19**). The isolation of **19** in yields ranging from 20–25% was achieved through reaction of **18** with fluoroacetic anhydride in the presence of triethylamine and catalytic amounts of 4-dimethylaminopyridine. However, it was noted that racemisation was unavoidable, and the method failed to produce the desired product for the valine analogue.

#### 2.1.4. Epoxide Ring Opening Approach with Fluoride Source

**Scheme 5:**
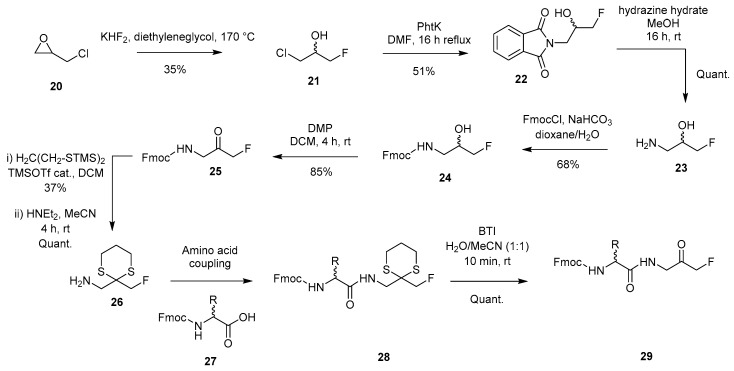
Synthesis of peptidyl FMK **29** utilising an epoxide ring opening approach [6].

The synthesis of peptidyl FMKs was also reported by Funeriu and coworkers in 2012. This was achieved through epoxide ring opening of epichlorohydrin **20** with a fluoride source (KHF_2_) to give **21**, which was then converted to *N*-phthalimid-fluoro-alcohol **22** in the presence of potassium phthalimide [6] (Scheme 5). Subsequent reaction with hydrazine allowed the generation of the free amine (**23**), which could then be Fmoc protected to give **24**, before oxidation with DMP-afforded FMK **25**. Temporary protection of the ketone functionality as a 1,3-dithiane followed by Fmoc deprotection was employed in order to allow peptide growth to occur. Regeneration of the ketone group was achieved using bis(trifluoroacetoxy)iodobenzene, giving peptidyl FMK **29** in a quantitative yield. A modified version of this procedure was utilised by Ellis et al. in 2016 in order to access peptidyl FMKs to be studied as irreversible protease inhibitors [8].

#### 2.1.5. Synthetic Pathway Involving Utilisation of a Fluorinated Hemiacetal/Aldehyde

**Scheme 6:**
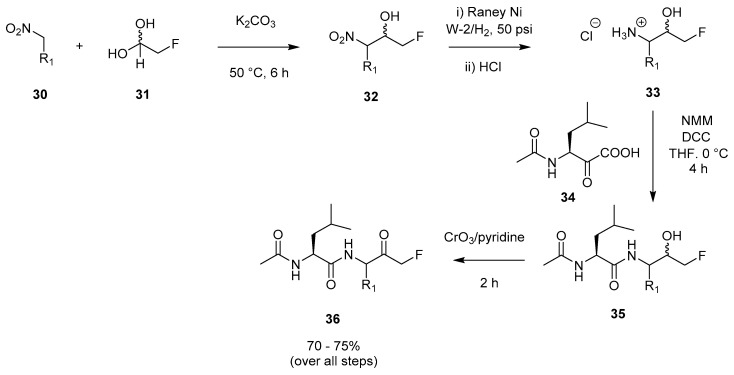
Isolation of peptidyl FMK **36** via a fluorinated hemiacetal [14].

The application of fluorinated hemiacetals/aldehydes has also been explored for accessing peptidyl FMKs. One particular example of this involves the formation of *β*-nitroalcohol species **32** derived from nitro alkane **30** reacting with a fluorinated hemiacetal (**31**), catalysed by potassium carbonate (Scheme 6) [14]. Further reduction with Raney nickel at 50 psi of hydrogen pressure followed by treatment with concentrated HCl allowed generation of **33** as a mixture of DL, *threo*, and *erythro* diastereomers. After liberation of the hydrochloride salt, peptide coupling with DCC as the activator afforded **35**. The final step in the process involved a Sarett oxidation, allowing isolation of FMK **36**. This pathway to **36** proceeded with an overall yield of 70–75%. A modified version of this method was employed by Revesz et al. in 1994 [27]. Chatterjee and coworkers described the preparation of peptidyl FMKs using a similar procedure [2], and also introduced a new approach which will be detailed in the next Section 2.1.6 [2].

**Scheme 7:**
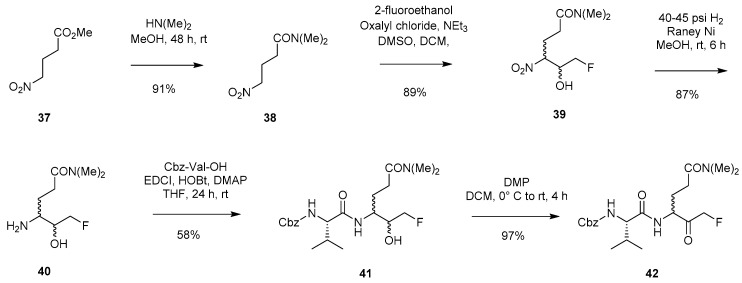
Synthetic route to peptidyl FMK **42** utilising a fluorinated aldehyde species [4].

The synthetic route presented by Cai et al. in 2006 (Scheme 7) closely resembles the methodology already discussed (Scheme 6), with the key difference being the use of a fluorinated aldehyde instead of the hemiacetal (**31**) [4]. Starting from ester **37**, conversion to amide **38** was achieved through the addition of dimethylamine. Further reaction with 2-fluoroacetaldehyde, which was accessed through Swern oxidation of the corresponding alcohol, enabled isolation of nitro-alcohol **39**. A subsequent hydrogenation step afforded **40** which could then be coupled to Cbz-Val-OH and finally oxidised to FMK **42**. Similar methodology was later employed by M. L. Forrest et al. in 2012 for accessing an FMK with a fluorescent moiety attached for cellular imaging purposes [7].

#### 2.1.6. Synthetic Methodology Employing Silyl Enol Ether Fluorination

**Scheme 8:**
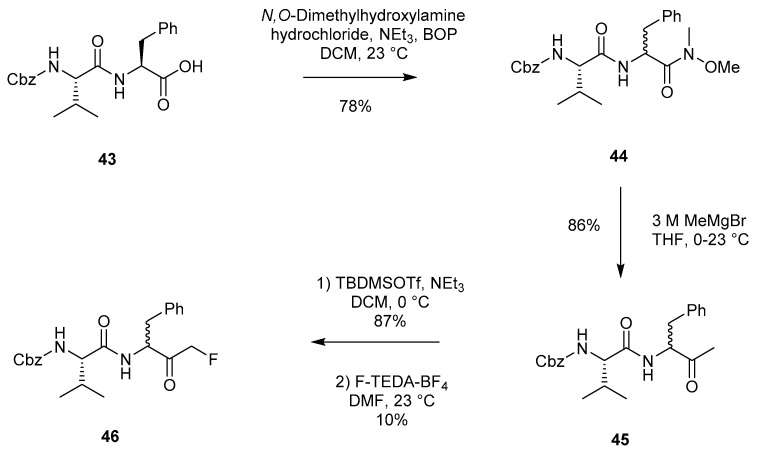
Isolation of dipeptide FMK **46** through silyl enol ether fluorination [2].

Chatterjee and coworkers described a novel approach for accessing peptidyl FMKs in 1997 (Scheme 8) [2]. Starting from Cbz-Val-Phe-OH (**43**), conversion to the Weinreb amide (**44**) was achieved with unavoidable epimerisation at the *P*_1_ position in the presence of *N,O*-dimethylhydroxylamine hydrochloride, triethylamine and BOP. Further reaction with MeMgBr led to the successful isolation of methyl ketone **45**. This could then be transformed into the corresponding silyl enol ether and subsequently fluorinated with F-TEDA-BF_4_, affording dipeptide FMK **46**.

#### 2.1.7. Synthetic Pathway Involving 1-amino-3-fluoro-propan-2-ol hydrochloride (**48**)

**Scheme 9:**
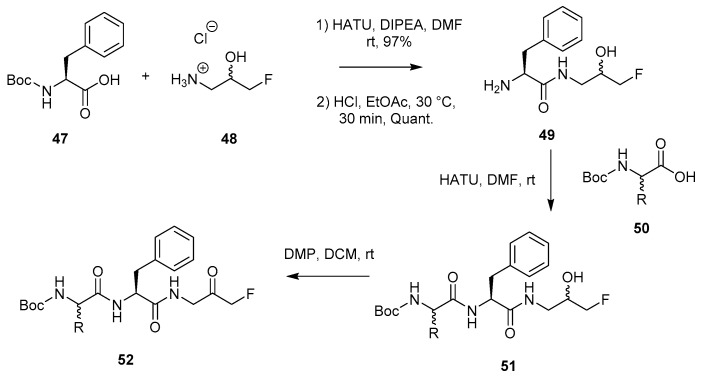
Formation of peptidyl FMK **52** starting from 1-amino-3-fluoro-propan-2-ol hydrochloride (**48**) [5].

A literature procedure for accessing peptidyl FMKs, which was reported by Tang et al. in 2016 (Scheme 9) [5], involves the coupling of a Boc-protected amino acid (**47**) with 1-amino-3-fluoro-propan-2-ol hydrochloride (**48**), which was synthesised according to a two-step procedure (not shown). Amide bond formation between **47** and **48** proceeds through exposure to HATU under basic conditions (DIPEA). Subsequent Boc deprotection of **49** is achieved by treatment with HCl in ethyl acetate, allowing for the coupling of the following amino acid. The final step in the process involves the oxidation of alcohol **51** to FMK **52** in the presence of Dess–Martin periodinane (DMP).

#### 2.1.8. Synthetic Approach Involving Employment of a Magnesium Fluoromalonate

**Scheme 10:**
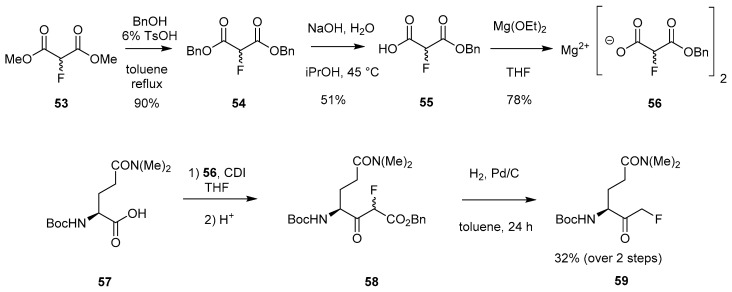
Synthesis of FMK **59** using magnesium benzyl fluoromalonate (**56**) [23].

In 1991, Palmer patented a novel approach to access peptidyl mono-FMKs through the use of magnesium fluoromalonate **56** [28]. This method was later employed by Vederas et al. in 1997, as shown in Scheme 10 [23]. After initial generation of magnesium benzyl fluoromalonate **56** from dimethyl fluoromalonate **53** (Scheme 10), further reaction with Boc-*N,N*-dimethylglutamine (**57**) in the presence of 1,1’-carbonyldiimidazole (CDI) afforded **58** without racemisation at the *P*_1_ position (Scheme 10). Subsequent catalytic hydrogenation gave the desired FMK **59**.

**Scheme 11:**
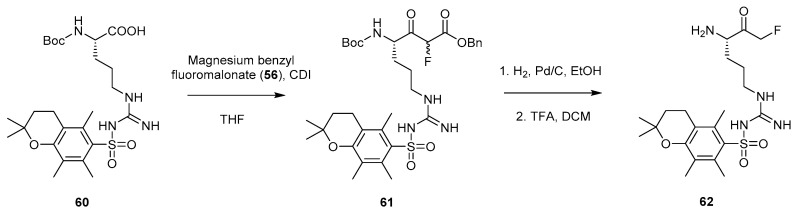
Synthesis of peptidyl FMK building block 62 using magnesium benzyl fluoromalonate (**56**) [3].

Implementation of this methodology was later carried out by Scott and coworkers in their work to explore the use of peptidyl FMKs as covalent inhibitors of MALT1 [3]. Magnesium benzyl fluoromalonate (**56**) was coupled to a Boc-arginine unit protected with a 2,2,5,7,8-pentamethylchroman (Pmc) sulfonyl group on the side chain (**60**) in the presence of CDI (Scheme 11). A one-pot benzyl ester deprotection and subsequent decarboxylation to the FMK was then achieved through the use of H_2_ and Pd/C in ethanol. A final selective Boc deprotection step gave the desired protected arginine building block (**62**) which could then be coupled to other amino acid units as required. Unfortunately, partial racemisation of the arginine residue was observed during the formation of **61**.

#### 2.1.9. Synthetic Approach Involving Magnesium Monobenzyl Fluoromalonate Enolate **65**

**Scheme 12:**
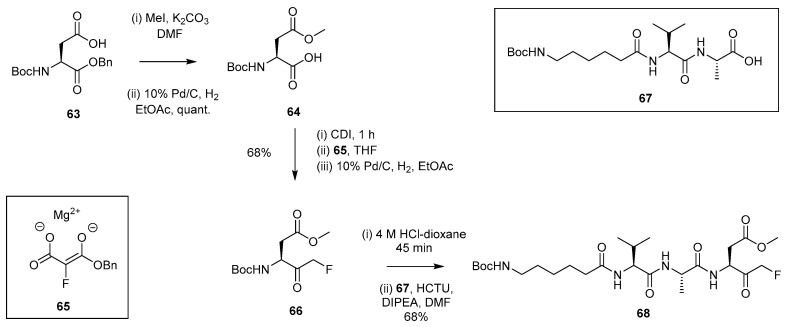
Synthetic route to peptidyl FMK **68** using fluorinated magnesium enolate **65** [29].

Van der Marel et al. [29] described an approach that made use of monobenzyl fluoromalonate magnesium enolate **65** as opposed to the neutral magnesium salt (**56**). The procedure for accessing FMK **68** (Scheme 12) involves an adapted version of the patented methodology described by Palmer et al. [30]. Initial side-chain esterification and subsequent debenzylation of **63** allowed isolation of acid **64**. Further reaction with CDI followed by addition of enolate **65** and subsequent hydrogenation gave FMK **66** in a yield of 68%. This was then Boc-deprotected under acidic conditions and coupled to fragment **67** in order to generate the FMK (**68**). In this case, the Boc group in **68** was removed and reacted with BODIPY TMR-OSu, which allowed for the preparation of a fluorescent probe for studying yeast peptide *N*-glycanase activity.

### 2.2. Solid-Phase Route

**Scheme 13:**
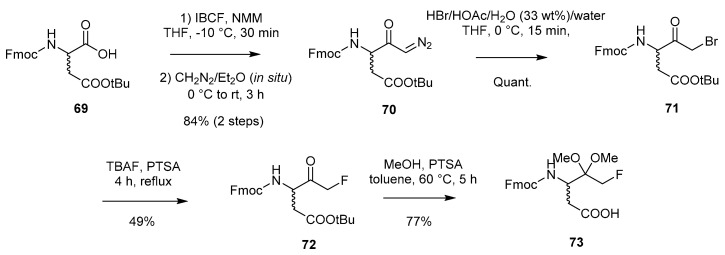
Generation of building block **73** from Fmoc-Asp(O*t*Bu)-OH (**69**) [31].

At present, only one solid-phase peptide synthesis (SPPS) method for accessing peptidyl mono-FMKs has been reported in the literature [31]. The approach involves initial formation of the FMK moiety through utilisation of a halogen-exchange reaction in a similar manner to the work of Kolb et al. [18] described earlier (Scheme 2). This is followed by attachment to the resin and peptide elongation. The first part of the process, involving construction of the FMK group from the amino acid building block Fmoc-Asp(O*t*Bu)-OH (**69**), is shown in Scheme 13. Firstly, the formation of diazoketone derivative **70** is achieved via a two-step process involving diazomethane. **70** is converted to the bromomethyl ketone **71** with HBr and then subsequently transformed into the desired FMK (**72**) using TBAF as a fluoride source in the presence of *p*-toluenesulfonic acid (PTSA). Repeating these steps using Fmoc-Gly-OH to produce the corresponding FMK analogue resulted in purification difficulties. As a result, the diazoketone was instead converted to the FMK via the hydroxymethyl ketone instead of the bromomethyl ketone, as this proved more successful for this particular substrate (not shown in scheme).

In the case of the FMK (**72**) obtained from Fmoc-Asp(O*t*Bu)-OH, because of the acid side-chain, attachment to the resin could occur in a straightforward manner, allowing for subsequent growth of the peptide chain and standard acidolytic cleavage from the resin. For use in solid-phase synthesis, temporary protection of the FMK ketone functionality was required. Thus, FMK **72** was heated for 5 h with methanol in the presence of PTSA to give dimethyl ketal **73** with concomitant removal of the *^t^*Bu side-chain protection.

As not all amino acids possess a carboxylic acid functional group in their side chain, an alternative approach needed to be employed for amino acids such as Gly. This modified approach involved the synthesis of a linker which could be anchored to both the ketone of the FMK using a 1,3-diol and to the resin via a carboxy group. The linker was made in such a way that it was stable during SPPS, yet capable of being cleaved under acidic conditions. Figure 2 shows the FMK moiety bound to the linker (**74**).

## 3. Applications and Uses of Peptidyl Mono-FMKs in Medicinal Chemistry

### 3.1. Drug Discovery

FMKs have shown potential for use in the development of drug therapeutics as well as chemical probes for interrogating cellular processes and the identification of protein targets [17,31]. In particular, FMKs have proven to be effective covalent protease inhibitors for the treatment of a wide range of diseases. These include rheumatoid arthritis, where FMKs with the ability to reduce the severity of inflammation and inhibit the extent of bone and cartilage damage have been reported [1,32,33]. Peptidyl FMKs have also shown significant promise as therapeutic agents for combating neurodegenerative disorders such as Alzheimer’s disease, along with various other illnesses including cancer [5]. Proteolytic cleavage of peptides within living organisms by protease enzymes is a vital process for maintaining optimum bodily function, with protease enzymes playing a significant role in many physiological processes including blood coagulation, digestion, and healing of wounds [34,35]. Despite this, if cleavage occurs in a disorderly and uncontrollable manner, the onset of ill health can be observed [36]. For this reason, the ability of certain inhibiting molecules to bind selectively to the proteases, preventing them from performing these disruptive cleavages, can prove hugely beneficial in the treatment of the patient. In addition to this, protease inhibitors can also be used for combating viral particles [37], as these too rely on proteolysis to be able to function properly. Peptidyl FMKs are capable of binding to these viral protease enzymes [38], blocking their activity and therefore preventing the maturation of infectious viral particles. This hinders replication, thus helping to combat disease.

#### 3.1.1. Peptidyl FMKs for the Treatment of Rheumatoid Arthritis

The use of peptidyl FMKs for the treatment of rheumatoid arthritis has been studied and subsequently reported in the literature [1]. FMKs have been designed that are able to inhibit cathepsin B, a cysteine protease, thus helping to reduce the extent of inflammation and combat cartilage and bone damage. The extracellular matrix of cartilage consists of proteoglycan and collagen molecules assembled in such a way that a rigid gel is formed, enabling joints to function properly. The development of arthritis has been associated with the release of cathepsins into inflamed tissues, causing destruction of the matrices because of proteoglycan and collagen degradation. This can have detrimental effects on the organism, leading to a loss of optimal joint function.

A selection of peptidyl FMK inhibitors, each consisting of a Phe-Ala moiety with variable *N*-terminal blocking groups, was synthesised and found to be effective irreversible inhibitors in vitro. The treatment of rats with a single oral dose of 25 mg/kg resulted in a 22–91% reduction in liver and kidney cathepsin B levels over a period of just 4 h (Table 1). The change in inhibition observed as a result of varying the *N*-terminal blocking group suggests that it has a significant effect on the interaction of the inhibitors with cathepsin B.

#### 3.1.2. Peptidyl FMKs for the Treatment of Neurological Disorders

The onset and progression of certain neurological diseases such as Alzheimer’s, stroke, and epilepsy has been associated with the presence of activated calpain I in the body [2]. During a stroke, the intracellular Ca^2+^ concentration increases as a result of various biochemical events. This subsequently leads to the activation of calpain I which is responsible for degradation of neuronal structural proteins. For this reason, the development of calpain I inhibitors is desirable in order to prevent neurodegeneration, thus combatting neurological disorders. A selection of peptidyl FMK dipeptides with varying amine protecting groups were synthesised and found to be potent irreversible inhibitors of calpain I. Interestingly, changing the *N*-capping group could be used to enhance the selectivity of the compound in favour of inhibiting calpain I over cathepsin B and L, along with altering potency. The absence of a protecting group was found to give poor inhibition, whilst *t*-Boc appeared to give the best selectivity for calpain I over cathepsin B. Additionally, a hydrophobic group was preferred at the *P*_1_ position in order for potent inhibition to occur. The importance of Leu or Val at *P*_2_ was also notable from the results reported. The most potent compound tested (*k* = 276,000 M^−1^ s^−1^) (**76**) along with the FMKs showing the greatest calpain I selectivity (**77** and **78**) can be seen in Figure 3. In addition to exhibiting good activity in an assay, these compounds were found to be cell permeable and capable of inhibiting intracellular recombinant human calpain I.

#### 3.1.3. Peptidyl FMKs as Inhibitors of MALT1 Paracaspase

The paracaspase enzyme MALT1 plays a key role in immune response through the activation of lymphocytes and other immune cells [3]. Despite the importance of these processes for healthy bodily function, if they occur in an abnormal manner, this can lead to the development of lymphoid malignancies. For this reason, the synthesis of inhibitors that target MALT1 proteases has proved an attractive method for the treatment of these tumours. Peptidyl-FMKs such as **79** (Figure 4) have shown potential for this purpose. The *P*1 Arg residue was found to be essential due to its interaction with the acidic residues in the target protein, whilst Leu at *P*4 helped by occupying a hydrophobic pocket. Although potent inhibitors have now been successfully developed for targeting MALT1 enzymes in vivo, the acquisition of orally available inhibitors remains an ongoing challenge.

#### 3.1.4. Dipeptidyl Glutaminyl FMKs as SARS-CoV Inhibitors

In 2003, an outbreak of SARS (Severe Acute Respiratory Syndrome) occurred as a result of the spread of coronavirus pathogen SARS-CoV. The virus, which leads to respiratory difficulties, was responsible for causing nearly 800 deaths; a value close to 10% of all confirmed cases in 2003. In 2006, S. X. Cai et al. synthesised a selection of dipeptidyl glutaminyl FMKs to be investigated as potential SARS-CoV inhibitors [4]. The viral cysteine protease enzyme M^pro^ [39] is essential for viral replication, and was thus identified as a possible drug target. Antiviral activity was evaluated through cytopathogenic effect (CPE) inhibition in SARS-CoV infected Vero and CaCo2 cultures. Cbz-Leu-Gln(NMe_2_)-FMK (**80a**) was found to be the most potent inhibitor, with an EC_50_ value of 2.5 µM in Vero cells (infected with strain 6109), low cellular toxicity, and a selectivity index of >40 (Table 2). FMKs **80b** and **80c** also showed some promising EC_50_ values (Table 2), and **80c** was also found to exhibit low toxicity in mice, suggesting that inhibitors should possess good safety profiles for further efficacy studies to be performed in animals.

### 3.2. Probes for Cellular Interrogation

**Figure 5:**
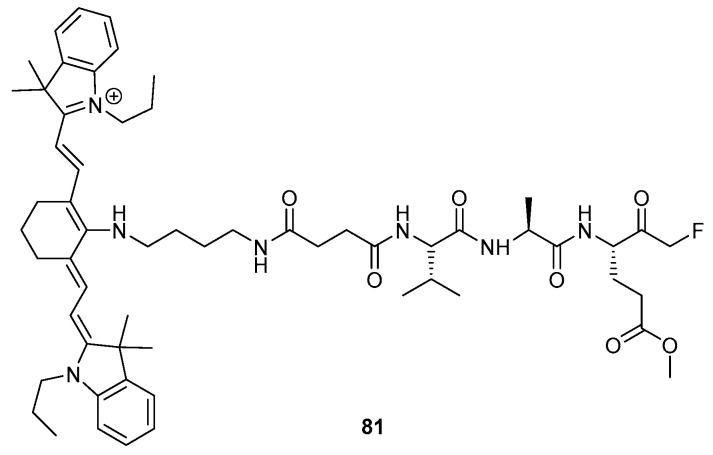
Structure of IR780-linker-Val-Ala-Glu(OMe)-FMK (**81**) [7].

In addition to their applications in the field of drug discovery, peptidyl FMKs have also been widely employed as chemical tools for the interrogation of biological systems [31]; for example, the elucidation of the structure and binding requirements for protease receptors in order to design drugs with greater selectivity and potency. Useful information can also be gained in relation to the active site selectivity of individual proteases within broader protease classes. This can be achieved through observing the nature of the binding interactions when amino acid groups in the peptide region of the peptidyl-FMK chain are varied until the binding requirements are fully satisfied [24]. Conjugation of dyes into the FMK structure, an example of which is shown in Figure 5 (**81**), has also been explored in order to allow for fluorescent imaging of cellular activity such as apoptosis [7].

**Figure 6:**
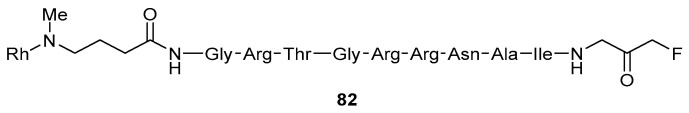
Peptidyl FMK possessing rhodamine B dye for studying PKACα activity [8].

Ellis and coworkers describe another example in which peptidyl FMKs are useful for understanding the structural binding requirements and properties of a target enzyme [8]. In this case, inhibitor **82** (Figure 6) was synthesised and found to be a potent and selective irreversible inhibitor of PKACα, a kinase enzyme. The structure of the inhibitor consists of an electrophilic fluoromethyl ketone moiety attached to a substrate-competitive inhibitor scaffold. This means that if the FMK binds in a region in which a reactive cysteine residue is present, the nucleophilic cysteine will attack the electrophilic FMK, causing inhibition. The nature of the peptide scaffold also plays a key role as it allows the peptide to bind in the vicinity of the reactive cysteine, an essential requirement for successful inhibition. As a result, inhibitor **82** is capable of covalently binding to and modifying a cysteine residue at the binding site of the enzyme. The incorporation of rhodamine B, a fluorescent tag, was employed in order to allow the inhibitor-substrate adduct to be observed, thus helping to confirm that the mechanism of inhibition occurs in an irreversible manner. In this way, the peptidyl FMK developed was found to be a useful tool for studying PKACα activity.

## 4. Conclusions

As highlighted, peptidyl mono-FMKs have been reported to exhibit promising therapeutic activity against a range of diseases including Rheumatoid arthritis, neurological diseases such as Alzheimer’s, lymphoid malignancies, and the SARS-CoV viral pathogen. Furthermore, FMKs can also be used as chemical probes for studying a range of cellular processes. The multifunctional nature of these biologically relevant FMK warheads, coupled with the fact that they are significantly more selective than the analogous chloromethyl-ketone-based inhibitors, makes them attractive moieties with potential utility in a wide range of applications including in vivo.

**Figure 7:**
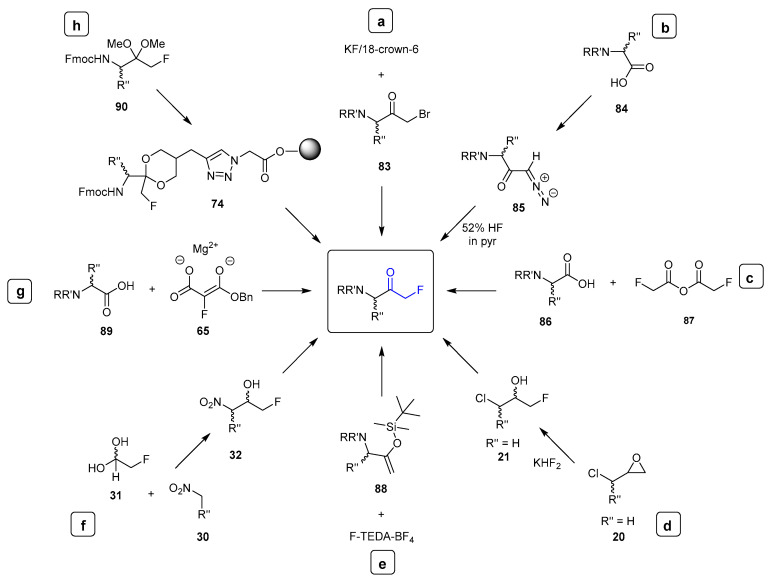
Summary of current solid- and solution-phase synthetic routes for accessing peptidyl mono-fluoromethyl ketones (FMKs).

Given their applications in these aforementioned areas, a range of synthetic approaches have been developed to access FMKs. The key synthetic solid- and solution-phase routes discussed in this review for accessing peptidyl mono-fluoromethyl ketones (FMKs) are summarised in Figure 7. These include halogen exchange methods (Figure 7a [22,23], diazomethyl ketone fluorination (Figure 7b) [24], a modified Dakin–West reaction (Figure 7c) [9], epoxide ring opening with a fluoride source (Figure 7d) [6,8], fluorination of silyl enol ethers (Figure 7e) [2], utilisation of a fluorinated hemiacetal/aldehyde (Figure 7f) [4,7,14,27], and the use of magnesium fluoromalonates (Figure 7g) [3,23,28,29,30]. Additionally, the use of a linker has been employed where appropriate for the synthesis of peptidyl FMKs by solid-phase peptide synthesis (Figure 7h) [31]. With many of these approaches suffering from low overall yields, racemisation, and incompatibility with solid phase peptide synthesis (SPPS) methodology, there is clearly still scope for the future development of new synthetic routes in this area.

## Figures and Tables

**Table 1:** % Inhibition of cathepsin B in liver and kidney tissue homogenates treated with 25 mg/kg of a selection of peptidyl FMKs [1].

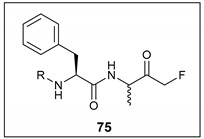					
R	% Inhibition in Liver Homogenates	% Inhibition in Kidney Homogenates	R	% Inhibition in Liver Homogenates	% Inhibition in Kidney Homogenates
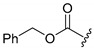	70.5	39.0	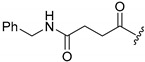	81.8	75.4
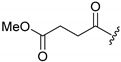	86.9	57.3	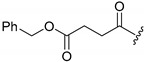	22.2	29.8
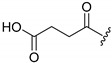	28.3	24.2	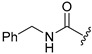	91.2	78.8

**Table 2:** EC_50_, CC_50_, and Selectivity Index (SI) values for a selection of dipeptidyl glutaminyl FMKs in SARS-CoV-infected Vero and CaCo2 cultures [4].

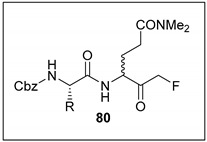							
			EC_50_ (µM)			CC_50_ (µM)	SI
			Vero		CaCo2	Vero	
Entry	Compound	R	FFM1	6109	FFM1		
1	**80a**	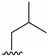	3.6 ± 1.3	2.5 ± 0.4	2.4 ± 0.56	>100	>40
2	**80b**	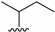	8.9 ± 2.9	5.3 ± 1.7	8.8 ± 2.5	>100	>18
3	**80c**	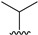	6.2 ± 1.9	6.6 ± 3.0	12.6 ± 4.1	>100	>15
